# Effectiveness and Safety of Acupuncture and Moxibustion for Primary Dysmenorrhea: An Overview of Systematic Reviews and Meta-Analyses

**DOI:** 10.1155/2020/8306165

**Published:** 2020-04-29

**Authors:** Jun Yang, Jun Xiong, Ting Yuan, Xue Wang, Yunfeng Jiang, Xiaohong Zhou, Kai Liao, Lingling Xu

**Affiliations:** ^1^Jiangxi University of TCM, Nanchang, Jiangxi, China; ^2^The Affiliated Hospital of Jiangxi University of TCM, Nanchang, Jiangxi, China

## Abstract

**Background:**

Acupuncture and moxibustion have been accepted as treatment options for primary dysmenorrhea (PD). So far, several systematic reviews (SRs) and meta-analyses (MAs) have reported on the efficacy and safety of acupuncture and moxibustion in treating PD.

**Objectives:**

The aim of this study was to critically summarize the evidence from relevant SRs and MAs reporting on the efficacy and safety of acupuncture and moxibustion in treatment of PD.

**Materials and Methods:**

Seven electronic databases, including Cochrane Database of Systematic Reviews, EMBASE, PubMed, SinoMed, China National Knowledge Infrastructure (CNKI), Chinese Science and Technology Periodical Database (VIP), and Wanfang database, were systematically searched. SRs or MAs about acupuncture for PD published up to May 2019 were included in the analysis. More than two authors independently assessed the quality of the evidence by AMSTAR2, PRISMA, PRISMA-A, and GRADE approach.

**Results:**

A total of 28 SRs and MAs, 281 original studies, reporting on 26,459 female patients were analyzed. The majority of the SRs were of moderate reporting quality and poor methodological quality. Moderate-quality evidence suggested that acupuncture and moxibustion were more effective compared to indomethacin or Fenbid in treating PD. Low-quality evidence suggested that, compared to NSAIDs, acupuncture and moxibustion could relieve pain with less adverse effects.

**Conclusion:**

Acupuncture and moxibustion seem to be effective and safe approaches in treatment of PD; yet, the methodological quality of most of the studies and the quality of evidence were low. Thus, additional studies are required to further confirm these results.

## 1. Introduction

Primary dysmenorrhea (PD) is a common gynecological disorder, mainly characterized by cramping pain in the lower abdomen that occurs before or during menstruation. Headache, nausea, vomiting, fatigue, irritability, diarrhea, and an overall feeling of discomfort are the common symptoms accompanying PD [1]. The disorder can significantly affect women's physical health and life quality [2]. In the USA, PD is responsible for the loss of 600 million work hours and two million dollars each year [3]. In China, the prevalence of dysmenorrhea is 30%∼80%, among which 53% is from PD and 15% from severe dysmenorrhea [4].

Nonsteroidal anti-inflammatory drugs (NSAIDs), oral contraceptive pills, or acupuncture are commonly used to alleviate the menstrual pain. Yet, drug therapy may lead to some adverse events, such as digestive disorders, headache, and drowsiness. In addition, 20%–25% of women do not respond well to these medications [5]. As nonpharmaceutical therapy, acupuncture stimulates the nervous system and release of endogenous substances, such as opioid peptides and serotonin, to improve symptoms [6, 7]. Moreover, moxibustion can regulate the levels of reproductive hormones to reduce the pain of dysmenorrhea [8]. Some studies have reported that the combination of acupuncture and moxibustion at SP6 could effectively improve the uterine artery hemodynamics and hemorheology in patients, as well as regulate blood viscosity and erythrocyte aggregation degree to relieve the pain [9–12].

Systematic reviews (SRs), especially those combined with meta-analyses (MAs), are of essential importance in evaluating clinical efficacy and formulating clinical guidelines [13, 14]. In their SR, Smith et al. have reported that current evidence cannot support the effectiveness of acupuncture in treating PD [15]. Moreover, Zhang et al. carried an overview to assess the effect of acupuncture and acupressure on PD using AMSTAR2 (A Measure Tool to Assess Systematic Reviews 2) and PRISMA (Preferred Reporting Items for Systematic Reviews and Meta-Analyses) tools [16–18]. They concentrated on acupuncture and acupressure, without referring to the moxibustion and quality of the available evidence. In addition, the results from SRs are conflicting, and the conclusions are limited. To overcome the limitations of an individual SR and to provide comprehensive evidence, an overview of multiple SRs, which is a new approach designed to synthesize the available results, is needed.

We enlarged the research source from seven databases and also considered the intervention of moxibustion so as to provide comprehensive evidence. Therefore, we conducted an overview to synthesize and critically appraise the available evidence on the effectiveness and safety of acupuncture and moxibustion for PD by using AMSTAR2, PRISMA, and GRADE (Grading of Recommendations Assessment, Development and Evaluation) approach [19].

## 2. Materials and Methods

### 2.1. Study Registration

The study has been registered in PROSPERO (http://www.crd.york.ac.uk/PROSPERO/display_record.php?ID=CRD42015016795).

### 2.2. Eligibility Criteria

#### 2.2.1. Types of Study

This SRs-MAs evaluate research articles written in Chinese and English reporting on effectiveness and safety of acupuncture and moxibustion for PD. Review articles, letters, conference papers, abstracts, protocols, and network meta analyses were excluded.

#### 2.2.2. Types of Participants

We included female patients of reproductive age suffering from PD. The definition of PD was based on cyclic pelvic pain during menstruation without any gynecological pathology, such as endometriosis, adenomyosis, or uterine myoma. Patients with secondary dysmenorrhea or serious medical conditions were excluded.

#### 2.2.3. Criteria for Intervention

The interventions included needle acupuncture, electro-acupuncture, auricular acupuncture, moxibustion, acupressure, point injection, or any combination of the above.

#### 2.2.4. Criteria for Comparison

We included western medicine, placebo, sham acupuncture, no treatment, or any combination of these.

#### 2.2.5. Primary Outcome

The total effective rate [20] was selected as a primary outcome. It was calculated based on the ratio of the total number of those who were effectively cured and the total number of sick people [20].

#### 2.2.6. Secondary Outcomes

Secondary outcomes were the following: clinical effective rate, visual analogue scale (VAS), adverse effects, quality of life, and symptom of dysmenorrhea. Clinical effective rate was calculated based on the ratio of the total number of people who responded well to therapy and the total number of sick people who continued to be sick [20]. VAS was analyzed according to a previously described approach [21]. Adverse effects were measured as incidence of side effects and types of side effects. Quality of life [22] was measured using a validation scale, for example, the Short Form (SF) 36. Symptoms of the dysmenorrhea were analyzed according to a previously described approach [23].

### 2.3. Search Strategy

We searched PubMed, Cochrane Database of Systematic Reviews, EMBASE, China National Knowledge Infrastructure (CNKI), Wanfang, Chinese Science and Technology Periodical Database (VIP), and sinoMed from inception to May 29, 2019. The following key search terms and their potential combination were used: “Acupuncture Analgesia”, “Acupuncture”, “Acupuncture Therapy”, “Acupuncture Points”, “moxibustion”, “primary dysmenorrhea”, “dysmenorrhea”, “systematic review”, and “meta-analysis”. Search strategies are shown in Table 1 or at the following link: http://www.crd.york.ac.uk/PROSPEROFILES/16795_STRATEGY_20150116.pdf.

### 2.4. Study Selection and Data Extraction

Two reviewers (JY and TY) separately searched the aforementioned databases and listed the titles of all articles. According to the inclusion criteria, by looking through the title and abstract, they excluded papers that were not eligible. Next, they screened the contents of the unclear articles further. If articles contained insufficient information to make a decision on eligibility, authors of the original reports were contacted so as to obtain further details. Finally, investigators (TY and XW) independently extracted data on the first author's name, year, studies/participants, intervention, comparison, main outcomes, and adverse effects from the full text, all of which were recorded by WPS 2019. Any disagreements were resolved by discussion or consulting with a third reviewer (XJ), until reaching a consensus.

### 2.5. Quality Assessment

On the basis of the first edition (AMSTAR), the newly developed high-quality evaluation tool of systematic review methodology (AMSTAR 2) has good consistency and practicability for estimators. We integrated the preferred reporting items for systematic reviews and Meta-Analyses-Abstract (PRISMA-A) and PRISMA to evaluate the reporting quality of the studies. The methodological quality and reporting quality of the included reviews were respectively assessed by AMSTAR2 and PRISMA.

The investigators systematically studied the relevant data and known evaluation methods. Two reviewers (YJ and YT) independently assessed the quality of the research; disagreements were solved by discussion or consulting with a third reviewer (JX). We calculated the number and 95% confidence intervals of 3 levels (“Yes,” “No,” or “Partial Yes”) for the AMSTAR-2 and PRISMA items. The 3 levels were scored as 1, 0.5, or 0 points separately for statistical analysis purposes. The methodological quality of each study was evaluated using the system evaluation credibility rating of AMSTAR 2.

### 2.6. Quality of Evidence

The GRADE approach was used to assess the quality of evidence for main outcomes. The rating included four levels: high, moderate, low, and very low, according to the quality of the evidence. Two reviewers (TY and XW) separately conducted the assessment process, any disagreement was resolved through discussion and consultation with a third author (JX) until a consensus was reached. An overview table, similar to a “summary of findings” table, was prepared with the help of GRADEPro software as per the GRADE approach. The summary table of the evidence for different SRs and MAs was prepared.

### 2.7. Strategy for Data Synthesis

We performed a re-meta-analysis of the data where two or more reviews reported on the same or similar intervention for outcomes relevant to our review. Given the overlap of some of SRs and MAs, two reviewers listed RCTs of each SRs and MAs and then excluded those that were overlapping. Risk indices (RRs) or odds ratios (ORs) were standardized for dichotomous outcomes; mean difference (MD) or standard mean difference (SMD) was used for continuous outcomes by using equations published in the Cochrane Handbook for Systematic Reviews of Interventions. RevMan5.3.5 software was used to calculate the standardized effect. According to the heterogeneity levels of the included SRs and meta-analyses, the random-effects model (*I*^2^ ≥ 50%) or fixed-effects model (*I*^2^ < 50%) was properly selected.

## 3. Results

### 3.1. Results on Literature Search and Selection

**Figure 1:**
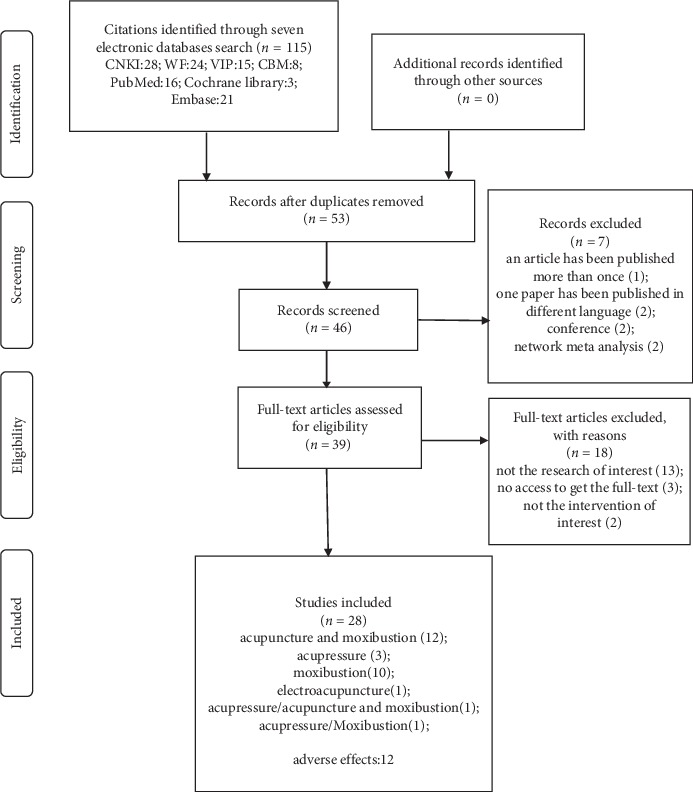
Flowchart of literature selection.

We obtained 115 relevant citations from seven electronic databases and manual searches. Before screening, 62 duplicates were excluded. After reading the title and abstract, seven records were rejected, including one article that has been published two times, three papers published in different languages, two conference papers, and 2 network meta-analyses. Full texts of the remaining 39 citations were retrieved for further assessment, and 18 citations were eliminated. Finally, 28 articles were included in this study. Exclusion list is described in additional file 1. The flowchart of literature selection is represented in Figure 1.

### 3.2. Characteristics of Included Reviews

Our analysis generated a total of 28 SRs and MAs, 281 original RCT or QRCT (Quasi-Randomized Controlled Trials) studies, including 26,459 patients of PD, published from 2009 to 2019. 12 [15.24.25.31.32.35.37.39.40.42.43.48] studies examined the effect of acupuncture and moxibustion, 3 [13.34.47] the effect of acupressure, 10 [14.26-30.33.36.38.41] the effect of moxibustion, 1 [24] electroacupuncture, 1 [15] acupressure/acupuncture and moxibustion, and 1 [25] acupressure/moxibustion.

Among these, 12 [15.24.26.30.31.36.39.41.42.43.47.48] reviews reported adverse effects; 16 reviews [13.14.15.25.26.29.31-33.36.38.40.44-46.48] and 10 reviews [24.27.28.34.35.37.39.42.43.47] applied Cochrane Handbook for Systematic Reviews of Interventions, Version 5.1.0, and Jadad scale for methodological quality assessment of original studies, respectively; and two reviews adopted double method to assess the treatment effect. As for main outcomes, 17 [13.14.24.25.27.28.30.33–38.40.41.46.47] took total effective rate as primary outcomes, 3 studies [26.29.39] payed more attention to clinical effective rate, 9 reviews [13.28.30.33.34.36.39.40.42] focused on VAS, and 12 studies [14.15.24.26.30.31.36.39.41.42.43.47] reported adverse effects. The characteristics of the literature search are shown in Table 2.

### 3.3. Methodological Assessment

We adopted AMSTAR 2 to assess the methodological quality of included studies (Table 3; additional file 2). The mean score was 8.8, ranging from 6 to 14. AMSTAR-2 score showed that the key factors affecting the quality of the literature included item 2 (2 studies explained their review methods before conducting the review), item 4 (1 study provided an comprehensive literature search strategy), item 7 (4 studies provided a list of excluded studies and justified the exclusions), item 9 (19 studies used a satisfactory technique for assessing RoB), item 11 (24 researches applied meta-analytical methods appropriately and gave explaining reasons; 2 studies did not provide the explanation of the heterogeneity), item 13 (25 studies accounted for RoB in individual studies in the results), and item 15 (22 studies took funnel plots or Egger's test and Begger's test to investigate the publication bias, and 9 of the studies gave explanation to discuss the potential impact on the results of the review).

Considering that the common problem of the included studies was lack of protocol and list of excluded studies, we adjusted the items 2 and 7 as the second line of the key factors in the process of the assessment. Our results revealed that more than half of the studies were graded as of critically low quality, 9 of low, 3 of moderate, and 1 of high quality.

Based on the quality results, most reviews followed the principle of PICO to carry on research and build framework. More than 2 reviewers performed study selection and extraction in duplicate. Reviewers evaluated the risk of bias of the included and providing satisfactory explanation for the results, while less attention was paid to the protocol and explanation for selection design, exclusion, or heterogeneity.

### 3.4. Reporting Quality

PRISMA and PRISMA-A were used to assess the reporting quality of studies (Table 4; additional file 3). The mean score was 28.45, ranging from 21.5 to 33. We found that most included reviews were of high reporting quality, with the part of the title, information sources, data collection process, risk of bias, and conclusion all being well reported. Some of the weaknesses of the reporting included lack to provide proper report in included studies, synthesis of the results, funding, and registration. In the method section, more than half of the studies provided search strategy for one database, while only one study provided a comprehensive literature search strategy. Thirteen studies did not make additional analysis, and twelve reviews did not refer to the funding.

### 3.5. Effectiveness of Acupuncture and Moxibustion

#### 3.5.1. Total Effective Rate

**Figure 2:**
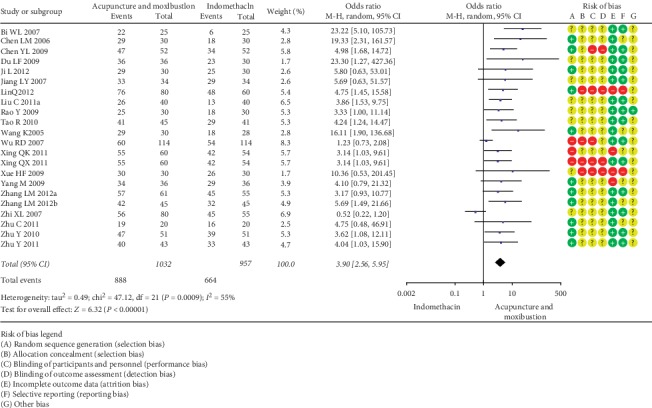
Acupuncture and moxibustion vs. indomethacin.

**Figure 3:**
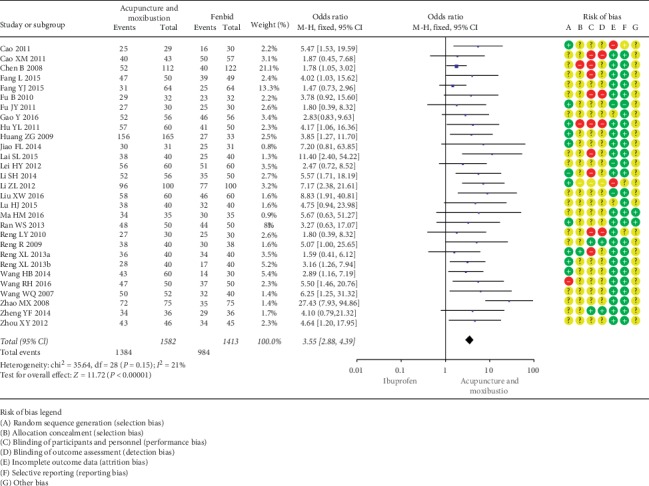
Acupuncture and moxibustion vs. ibuprofen.

**Figure 4:**
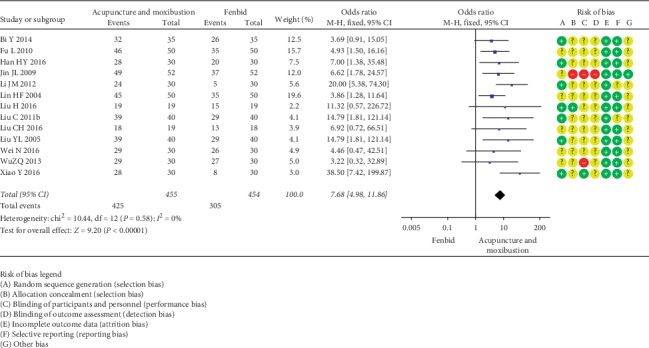
Acupuncture and moxibustion vs. Fenbid.

**Figure 5:**
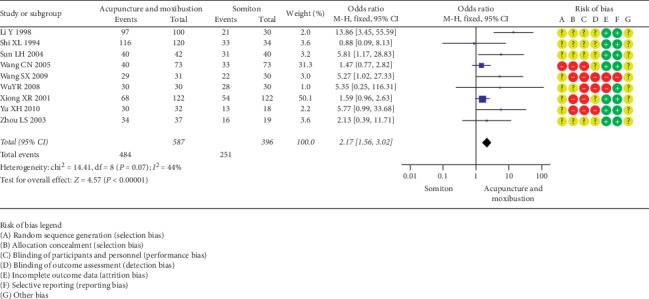
Acupuncture and moxibustion vs. Somiton.

*(1) Acupuncture and Moxibustion vs. Indomethacin/Ibuprofen/Fenbid/Somiton*. Sixteen SRs [13.14.15.24.25.26.28.30.33.37.39.40.42.43.45.48] encompassing 22 RCTs (1989 participants) suggested that a *combination* of acupuncture and moxibustion was superior to indomethacin in treating PD (OR = 3.9, 95% CI (2.56, 5.95; *P* < 0.00001; Figure 2). Furthermore, 17 SRs [14.15.25-30.35.37.39.40-43.45.46], including 29 RCTs (2995 participants), suggested that a combination of acupuncture and moxibustion was more effective than ibuprofen for treatment of PD (OR = 3.55, 95% CI (2.98, 4.39); *P* < 0.00001; Figure 3). Moreover, 12 SRs encompassing [13.15.24.35.37–40.42.43.45.46] 13 RCTs (909 participants) showed that acupuncture and moxibustion were superior to Fenbid (OR = 7.68, 95% CI (4.98, 11.86); *P* < 0.00001; Figure 4). Also, 5 SRs [14.25.40.42.43] covering 9 RCTs (983 participants) showed that acupuncture and moxibustion were superior to Somiton in treatment of PD patients (OR = 2.17, 95% CI (1.56, 3.02); *P* < 0.00001; Figure 5).

#### 3.5.2. VAS

**Figure 6:**
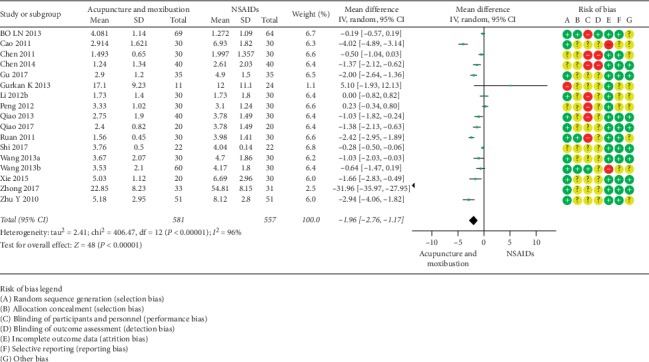
Acupuncture and moxibustion vs. NSAIDs.

**Figure 7:**
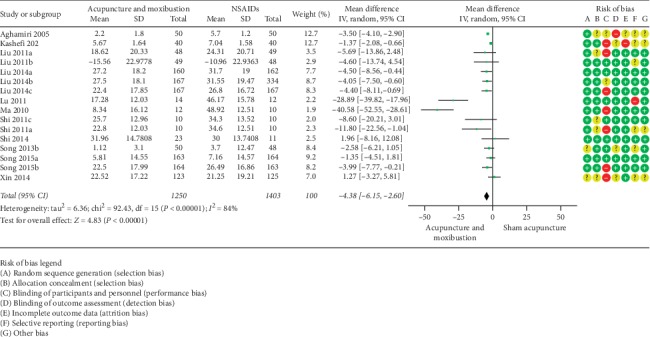
Acupuncture and moxibustion vs. sham acupuncture.

**Figure 8:**
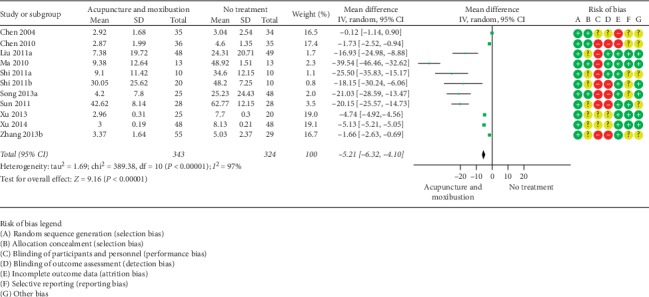
Acupuncture and moxibustion vs. no treatment.

*(1) Acupuncture and Moxibustion vs. NSAIDs/Sham Acupuncture/No Treatment*. Seven SRs [13.15.22.32.43.47.48] encompassing 17 RCTs (1138 participants) suggested that a combination of acupuncture and moxibustion was superior to NSAIDs in relieving pain (MD = −1.96, 95% CI (−2.76, −1.17); *P* < 0.00001; Figure 6). In addition, 5 SRs [15.31.32.44.45] encompassing 16 RCTs (2653 participants) reported that acupuncture and moxibustion significantly reduced the pain compared with sham acupuncture (MD = −4.38, 95% CI (−6.15, −2.60); *P* < 0.00001; Figure 7). Moreover, four SRs [15.31.44.45] encompassing 11 RCTs (667 participants) consistently showed that acupuncture and moxibustion were superior to no treatment in relieving pain MD = −5.21 95% CI (−6.32, −4.10); *P* < 0.00001; Figure 8).

### 3.6. Adverse Events

**Figure 9:**
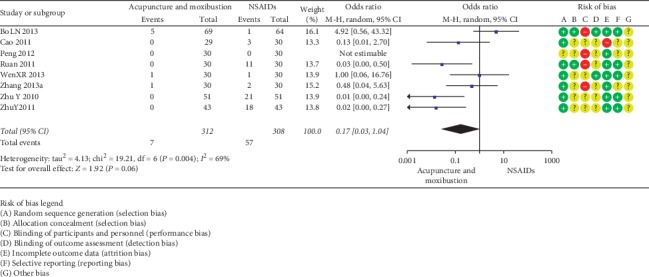
Acupuncture and moxibustion vs. NSAIDs.

Seven SRs [13.15.24.30.36.38.47] encompassing 8 RCTs (667 participants) consistently showed that acupuncture and moxibustion were safer compared to NSAIDs in treatment of PD (OR = 0.17, 95% CI (0.03, 1.04); *P*=0.06; Figure 9).

### 3.7. Quality of Evidence

The quality of evidence for 3 outcomes (total effective rate, VAS and adverse events) is shown in Table 5. The results showed that the quality of the evidence was low and all the outcomes were biased in allocation concealment or inadequate blinding; the outcomes of the VAS and adverse events were inconsistent, which was caused by course or treatment of the patient. The funnel plot of the total effective rate (acupuncture and moxibustion vs. indomethacin and acupuncture and moxibustion vs. Somiton) and VAS (acupuncture and moxibustion vs. no treatment) was dissymmetrical.

## 4. Discussion

### 4.1. Summary of Main Findings

This overview provided a comprehensive overview of the evidence on the effectiveness and safety of acupuncture and moxibustion for PD. Evidence of moderate quality suggested that acupuncture and moxibustion had a positive effect on indomethacin or Fenbid in treating PD. Low-quality evidence showed that compared to NSAIDs, acupuncture and moxibustion could relieve PD related pain with less adverse effects, which needs to be further researched. The adverse effects related to the acupuncture and moxibustion were mild, and they included dizziness, fainting, or minimal bleeding after acupuncture.

The majority of the SRs were of moderate reporting quality and poor methodological quality. Most of the studies followed the principle of PICO to carry on research and build framework, select proper assessment tool or appropriate methods for statistical combination of results, while they fail to provide registration, and assess the potential impact of individual ROB studies on the results of the meta-analysis or other evidence synthesis. Most of the RCTs did not explain the treatment allocation concealed and blinding. The quality of the reporting of the SRs was limited by lack of data on registration and funding, comprehensive search strategy, and explanation of the heterogeneity. With reference to the abstract, although many studies reported structured abstract, they failed to fully report the synthesis of results, the risk of basis, funding, and registration.

### 4.2. Strengths and Limitation

Following is the brief summary of the present research: (1) comprehensive search strategies were applied to seven databases to ensure that all relevant reviews were identified; (2) before assessment, we have systematically learned some related courses on methodology and reporting evaluation and consulted relevant methodological experts, professors so as to gain deep understanding, and ensure the accuracy of the evaluation process; (3) during the process, we adopted AMSTAR 2 and PRISMA to evaluate the methodological and reporting quality of the qualified studies, and we combined PRISMA-A to the part of abstract in PRISMA, thus making the evaluation more precise; (4) we excluded the overlapping RCTs and conducted a quantitative analysis of the primary RCTs, with the help of the GRADE approach so as to evaluate the quality of the outcomes with different comparisons; (5) Cochrane Collaboration guidelines were followed for data synthesis. More than two reviewers were engaged so as to minimize potential bias in the overview process.

There are some limitations in the present study: (1) The methodological quality of both included SRs and primary RCTs was not high, and the quality of evidence for the outcomes was unsatisfactory; thus, the conclusions from this overview should be interpreted with caution. (2) The current overview was constrained by limitations of the included SRs. During the process of literature selection, some SRs and MAs included Q-RCT (quasi-randomized controlled trials). The increase of complex factors led to less reliance in our overview. (3) We collected evidence on acupuncture and moxibustion for PD, while we failed to separate different types of acupuncture interventions.

### 4.3. Opportunities for Future Research

Through this overview, we found that current evidence is of low quality; hence, further research is needed. (1) High-quality RCTs with large sample sizes are necessary to demonstrate the safety of different types of acupuncture interventions for PD. (2) RCTs or SRs should follow the corresponding guidelines in their reporting. CONSORT (Consolidated Standards of Reporting Trials) [49] are applied to all kinds of RCTs, and they include some characteristic guidelines such as STRICTA (Standards for Reporting Interventions in Controlled Trials of Acupuncture) [50] and STRICTOM (The Standards for Reporting Interventions in Clinical Trials Of Moxibustion) [51] for acupuncture and moxibustion, independently. PRISMA is used for SR and MA. (3) We also recommend GRADE approach to assess the evidence quality of the more SRs in the future. (4) The primary RCTs should give more attention to blinding, allocation concealment, and registration, which could result in more reliable evidence.

## 5. Conclusion

In conclusion, the current evidence suggests that acupuncture and moxibustion is more effective than ibuprofen or Fenbid in the treatment of PD. While there is no enough evidence to support that acupuncture and moxibustion are safe methods to relieve pain and improve the VAS, future studies should place more emphasis on the safety of acupuncture for PD. Also, more efforts are required to improve the study quality of RCTs and SRs, and researchers should strictly adhere to the CONSORT and PRISMA guidelines.

## Figures and Tables

**Table 1:** Search strategy (PubMed).

Order	Strategy
#1	Search “dysmenorrhea”[Mesh]
#2	Search (((primary dysmenorrhea[Title/Abstract]) OR PD[Title/Abstract]) OR dysmenorrhea[Title/Abstract])
#3	#1 OR #2
#4	Search “Acupuncture”[Mesh] OR “Acupuncture Therapy”[Mesh] OR “Acupuncture, Ear”[Mesh] OR “Acupuncture Points”[Mesh] OR “Acupuncture Analgesia”[Mesh] OR “moxibustion”[Mesh]
#5	Search (((((Acupuncture[Title/Abstract]) OR Acupuncture Therapy[Title/Abstract]) OR Acupuncture, Ear[Title/Abstract]) OR Acupuncture Points[Title/Abstract]) OR acupoint[Title/Abstract]) OR electropuncture[Title/Abstract] OR “moxibustion”[Title/Abstract])
#6	#4 OR #5
#7	Search “Systematic Reviews as Topic”[Mesh] OR “Systematic Review”[Publication Type]
#8	Search (((((Systematic Reviews[Title/Abstract]) OR Systematic Review[Title/Abstract]) OR SR[Title/Abstract]) OR SRs[Title/Abstract]) OR Review[Title/Abstract])
#9	Search (systematic[Title/Abstract]) AND review[Title/Abstract]
#10	Search “Meta-Analysis”[Publication Type] OR “Meta-Analysis as Topic”[Mesh]
#11	Search ((Meta-Analysis[Title/Abstract]) OR meta-analysis[Title/Abstract]) OR meta‐analy^∗^[Title/Abstract]
#12	#7 OR #8 OR #9 OR #10 OR #11
#13	#3 AND #6 AND #12

**Table 2:** Characteristics of the literature search.

Review	Studies (participants)	Intervention	Comparison	Main outcomes	Risk assessment tool	Adverse effect
Fan [26]	14 (1320)	Acupuncture and moxibustion	Western medicine/Chinese medicine	Total effective rate, VAS, adverse effects	Jadad	Y
Lan et al. [27]	7 (822)	Acupuncture and moxibustion/Acupuncture and moxibustion + others	Placebo acupuncture/Western medicine/blank	Total effective rate	RoB	N
Chen et al. [28]	14 (1320)	Heat-sensitive moxibustion	No limit	Clinical effective rate	RoB	Y
Xu et al. [29]	15 (1261)	Moxibustion/Moxibustion + others	Not moxibustion	Total effective rate	Jadad	N
Wang et al. [30]	12 (957)	Heat sensitive moxibustion/Heat sensitive moxibustion + others	No limit	Total effective rate, symptom score of the dysmenorrhea	Jadad	N
Zhou et al. [31]	7 (542)	Heat sensitive moxibustion	No limit	Clinical effective rate, cure rate, CMSS	RoB	N
Lu et al. [32]	13 (1524)	Indirect moxibustion	Western medicine/Chinese medicine	Total effective rate, symptom of the dysmenorrhea, adverse effects	Jadad, RoB	Y
Woo et al. [33]	60 (5901)	Acupuncture and moxibustion	Western medicine/sham acupuncture/blank	Pain intensity, pain relief、SF-36	RoB	Y
Tong et al. [34]	23 (2770)	Acupuncture and moxibustion	Sham acupuncture	VAS, VRS, NRS	RoB	N
Fan et al. [35]	13 (1040)	Warm needling method	Western medicine/Chinese medicine	Total effective rate, symptom score of the dysmenorrhea	RoB	N
Sun et al. [36]	8 (644)	Acupressure/acupressure + others	Acupuncture + others	Symptom score of the dysmenorrhea, total effective rate, VAS	Jadad	N
Qin et al. [37]	19 (1760)	Acupuncture and moxibustion	Western medicine/Chinese medicine	Total effective rate	Jadad	N
Gou [38]	10 (586)	Moxibustion	Not moxibustion	Total effective rate, symptom of the dysmenorrhea	RoB	Y
Liu et al. [39]	14 (1123)	Acupuncture and moxibustion/acupuncture and moxibustion + Western medicine/acupuncture and moxibustion + Chinese medicine	Western medicine/Chinese medicine	Total effective rate	Jadad	N
Gou et al. [40]	12 (786)	Moxibustion	Not moxibustion	Total effective rate pain	RoB	Y
Wang [12]	12 (1236)	Indirect moxibustion	Western medicine/Chinese medicine	Total effective rate	RoB	N
Lin et al. [41]	15 (1594)	Acupuncture and moxibustion	Western medicine/Chinese medicine	Clinical effective rate, symptom score of the dysmenorrhea	Jadad	N
Qin et al. [42]	20 (2134)	Acupuncture and moxibustion	Western medicine/Chinese medicine	Total effective rate, symptom score of the dysmenorrhea	RoB	N
Listijo [43]	11 (412)	Moxibustion	Western medicine/Chinese medicine/acupuncture	Total effective rate	Jadad, RoB	Y
Chen et al. [44]	28 (2787)	Acupuncture and moxibustion/Acupuncture and Moxibustion + others	Western medicine/Chinese medicine	Total effective rate, symptom score of the dysmenorrhea	Jadad	Y
Yang [45]	32 (3910)	Acupuncture and moxibustion	No treatment/placebo/acupressure/Western medicine/Chinese medicine	VAS	Jadad	Y
Chen et al. [46]	8 (589)	Acupressure/Acupuncture and moxibustion	Acupuncture/sham acupuncture	VAS	RoB	N
Yu et al. [24]	9 (3118)	Electroacupuncture	Pharmacological treatment/nonacupoints/waiting-list groups	VAS, RSS	RoB	N
Xu et al. [11]	16 (1679)	Acupoint-stimulation	NSAIDs	Total effective rate, symptom score of the dysmenorrhea	RoB	N
Smith et al. [15]	42 (4640)	Acupressure/Acupuncture and moxibustion	NSAIDs/placebo/blank	VAS	RoB	Y
Xu et al. [25]	20 (2134)	Acupressure/moxibustion	Not acupuncture and moxibustion	Total effective rate, pain intensity	RoB	N
Chung et al. [47]	25 (3109)	Acupoint stimulation	No limit	Total effective rate, adverse effects	Jadad	Y
Cho and Hwang [48]	27 (2806)	Acupuncture	No limit	Pain relief	RoB	Y

Y: yes; N: no.

**Table 3:** Methodological quality of the included reviews assessed by AMSTAR2

Item no.	Checklist item	Y		PY		N	
		*n* (%)	95% CI	*n* (%)	95% CI	*n* (%)	95% CI
1	Did the research questions and inclusion criteria for the review include the components of PICO (population, intervention, control group, and outcome)?	27 (96.43)	[0.89, 1.03]	0		1 (3.57)	[−0.03, 0.10]
2	Did the report of the review contain an explicit statement that the review methods were established prior to the conduct of the review and did the report justify any significant deviations from the protocol?	2 (7.14)	[−0.02, 0.17]	0		26 (92.86)	[0.83, 1.02]
3	Did the review authors explain their selection of the study designs for inclusion in the review?	0		0		28 (100)	
4	Did the review authors use a comprehensive literature search strategy?	1 (3.57)	[−0.03, 0.10]	27 (96.43)	[0.89, 1.03]	0	
5	Did the review authors perform study selection in duplicate?	26 (92.86)	[0.83, 1.02]	0		2 (7.14)	[−0.02, 0.17]
6	Did the review authors perform data extraction in duplicate?	25 (89.29)	[0.78, 1.01]	0		3 (10.71)	[−0.01, 0.22]
7	Did the review authors provide a list of excluded studies and justify the exclusions?	4 (14.29)	[0.01, 0.27]	1 (3.57)	[−0.03, 0.10]	23 (82.14)	[0.68, 0.96]
8	Did the review authors describe the included studies in adequate detail?	3 (10.71)	[−0.01, 0.22]	25 (89.29)	[0.78, 1.01]	0	
9	Did the review authors use a satisfactory technique for assessing the risk of bias (RoB) in individual studies that were included in the review?	19 (67.86)	[0.51, 0.85]	9 (32.14)	[0.15, 0.49]	0	
10	Did the review authors report on the sources of funding for the studies included in the review?	15 (53.57)	[0.35,0.72]	0		13 (46.43)	[0.28, 0.65]
11	If meta-analysis (MA) was justified did the review authors use appropriate methods for statistical combination of results?	24 (85.71)	[0.73, 0.99]	2 (7.14)	[−0.02, 0.17]	2 (7.14)	[−0.02, 0.17]
12	If meta-analysis was performed did the review authors assess the potential impact of RoB in individual studies on the results of the meta-analysis or other evidence synthesis?	4 (14.29)	[0.01, 0.27]	0		24 (85.71)	[0.73, 0.99]
13	Did the review authors account for RoB in individual studies when interpreting/discussing the results of the review?	25 (89.29)	[0.78, 1.01]	0		3 (10.71)	[−0.01, 0.22]
14	Did the review authors provide a satisfactory explanation for, and discussion of, any heterogeneity observed in the results of the review?	23 (82.14)	[0.68, 0.96]	1 (3.57)	[−0.03, 0.10]	5 (17.86)	[0.04, 0.32]
15	If they performed quantitative synthesis did the review authors carry out an adequate investigation of publication bias (small study bias) and discuss its likely impact on the results of the review?	9 (32.14)	[0.15, 0.49]	13 (46.43)	[0.28, 0.65]	6 (21.43)	[0.06, 0.37]
16	Did the review authors report any potential sources of conflict of interest, including any funding they received for conducting the review?	12 (42.86)	[0.25, 0.61]	2 (7.14)	[−0.02, 0.17]	14 (50)	[0,31, 0.69]

Y: yes; N: no; P: partial satisfaction.

**Table 4:** Reporting quality of the included reviews assessed by PRISMA.

Item	Checklist item PRISMA	Y		PY		N	
		*n* (%)	95% CI	*n* (%)	95% CI	*n* (%)	95% CI
Tiltle					
	Title	27 (96.43)	[0.89, 1.03]	0		1 (3.57)	[−0.03, 0.10]
Abstract					
	Objectives	28 (100)		0		0	
	Eligibility criteria	28 (100)		0		0	
	Information sources	27 (96.43)	[0.89, 1.03]	0		1 (3.57)	[−0.03, 0.10]
	Risk of bias	7 (25)	[0.09, 0.41]	0		21 (75)	[0.59, 0.91]
	Included studies	23 (82.14)	[0.68, 0.96]	0		5 (17.86)	[0.04, 0.32]
	Synthesis of results	2 (7.14)	[−0.02, 0.17]	18 (64.29)	[0.47, 0.82]	8 (28.57)	[0.12, 0.45]
	Description of the effect	19 (67.86)	[0.51, 0.85]	0		9 (32.14)	[0.15, 0.49]
	Strengths and limitations of evidence	22 (78.57)	[0.63, 0.94]	0		6 (21.43)	[0.06, 0.37]
	Interpretation	28 (100)		0		0	
	Funding	0		0		28 (100)	
	Registration	1 (3.57)	[−0.03, 0.10]	0		27 (96.43)	[0.89, 1.03]
Introduction
	Rationale	27 (96.43)	[0.89, 1.03]	0		1 (3.57)	[−0.03, 0.10]
	Objectives	28 (100)		0		0	
Methods
	Protocol and registration	4 (14.29)	[0.01, 0.27]	0		24 (85.71)	[0.73, 0.99]
	Eligibility criteria	27 (96.43)	[0.89, 1.03]	1 (3.57)	[−0.03, 0.10]	0	
	Information sources	28 (100)		0		0	
	Search	7 (25)	[0.09, 0.41]	21 (75)	[0.59, 0.91]		
	Study selection	24 (85.71)	[0.73, 0.99]	2 (7.14)	[−0.02, 0.17]	2 (7.14)	[−0.02, 0.17]
	Data collection process	25 (89.29)	[0.78, 1.01]	1 (3.57)	[−0.03, 0.10]	2 (7.14)	[−0.02, 0.17]
	Data items	19 (67.86)	[0.51, 0.85]	2 (7.14)	[−0.02, 0.17]	7 (25)	[0.09, 0.41]
	Risk of bias in individual studies	13 (46.43)	[0.28, 0.65]	15 (53.57)	[0.35, 0.72]	0	
	Summary measures	28 (100)					
	Synthesis of results	25 (89.29)	[0.78, 1.01]	0		3 (10.71)	[−0.01, 0.22]
	Risk of bias across studies	21 (75)	[0.59, 0.91]	4 (14.29)	[0.01, 0.27]	3 (10.71)	[−0.01, 0.22]
	Additional analyses	17 (60.71)	[0.43, 0.79]	0		11 (39.29)	[0.21, 0.57]
Results
	Study selection	27 (96.43)	[0.89, 1.03]	0		1 (3.57)	[−0.03, 0.10]
	Study characteristics	27 (96.43)	[0.89, 1.03]	1 (3.57)	[−0.03, 0.10]	0	
	Risk of bias within studies	27 (96.43)	[0.89, 1.03]	0		1 (3.57)	[−0.03, 0.10]
	Results of individual studies	28 (100)		0		0	
	Synthesis of results	26 (92.86)	[0.83, 1.02]	1 (3.57)	[−0.03, 0.10]	1 (3.57)	[−0.03, 0.10]
	Risk of bias across studies	27 (96.43)	[0.89, 1.03]	1 (3.57)	[−0.03, 0.10]	0	
	Additional analysis	15 (53.57)	[0.35, 0.72]	0		13 (46.43)	[0.28, 0.65]
Discussion
	Summary of evidence	8 (28.57)	[0.12, 0.45]	20 (71.43)	[0.55, 0.88]	0	
	Limitations	26 (92.86)	[0.83, 1.02]	0		2 (7.14)	[−0.02, 0.17]
	Conclusions	26 (92.86)	[0.83, 1.02]	1 (3.57)	[−0.03, 0.10]	1 (3.57)	[−0.03, 0.10]
Funding
	Funding	10 (35.71)	[0.18, 0.53]	6 (21.43)	[0.06, 0.37]	12 (42.86)	[0.25, 0.61]

Y: yes; N: no; P: partial satisfaction.

**Table 5:** Quality of evidence in the included studies assessed by the GRADE approach.

Outcome	Intervention vs. comparison	Included studies	Effect size	Quality of the evidence
Total effective rate	Acupuncture and moxibustion vs. indomethacin	16 SRs, 22 RCTs	OR = 3.9, 95% CI (2.56, 5.95)	⊕ ◯ ◯ ◯(1).(2).(3) very low
	Acupuncture and moxibustion vs. ibuprofen	17 SRs, 29 RCTs	OR = 3.55, 95% CI (2.88, 4.39)	⊕ ⊕ ⊕ ◯(1) moderate
	Acupuncture and moxibustion vs. Fenbid	12 SRs, 13 RCTs	OR = 7.68, 95% CI (4.98, 11.86)	⊕ ⊕ ⊕ ◯(1) moderate
	Acupuncture and moxibustion vs. Somiton	5 SRs, 9 RCTs	OR = 2.17, 95% CI (1.56, 3.02)	⊕ ⊕ ◯⃝(1).(3) low
VAS	Acupuncture and moxibustion vs. NSAIDs	7 SRs, 17 RCTs	MD = −1.96, 95% CI (−2.76, −1.17)	⊕ ⊕ ◯ ◯(1).(2) low
	Acupuncture and moxibustion vs. sham acupuncture	5 SRs, 16 RCTs	MD = −4.38, 95% CI (−6.15, −2.60)	⊕ ⊕ ◯ ◯(1).(2) low
	Acupuncture and moxibustion vs. no treatment	4 SRs, 11 RCTs	MD = −5.21, 95% CI (−6.32, −4.10)	⊕ ◯ ◯ ◯(1).(2).(3) very low
Adverse events	Acupuncture and moxibustion vs. NSAIDs	7 SRs, 8 RCTs	OR = 0.17, 95% CI (0.03, 1.04)	⊕ ⊕ ◯ ◯(1).(2) low

(1) Allocation concealment or blinding inadequate; (2) *I*^2^ > 50% or large heterogeneity; (3) funnel plot dissymmetry or language limitation. ⊕: +1, ◯: −1, ⊕ ⊕ ⊕ ⊕ : High, ⊕ ⊕ ⊕ ◯ : Moderate, ⊕ ⊕ ◯ ◯ : Low, and ⊕ ◯ ◯ ◯ : very low.

## Abbreviations

PD: Primary dysmenorrhea
SR: Systematic review
MA: Meta-analysis
CNKI: China National Knowledge Infrastructure
VIP: Chinese Science and Technology Periodical Database
AMSTAR2: A measure tool to assess systematic reviews 2
PRISMA: Preferred Reporting Items for Systematic Reviews and Meta-Analyses
PRISMA-A: Preferred Reporting Items for Systematic Reviews and Meta-Analyses-Abstract
GRADE: Grading of Recommendations Assessment, Development, and Evaluation
CONSORT: Consolidated Standards of Reporting Trials
STRICTA: Standards for Reporting Interventions in Controlled Trials of Acupuncture
STRICTOM: The Standards for Reporting Interventions in Clinical Trials of Moxibustion
VAS: Visual analogue scale
NSAIDs: Nonsteroidal anti-inflammatory drugs.

## Supplementary Materials

Supplementary Materials Additional file 1: exclusion list; additional file 2: AMSTAR 2 evaluation of acupuncture and moxibustion for PD; additional file 3: PRISMA evaluation of acupuncture and moxibustion for PD; additional file 4: risk of bias summary; additional file 5: risk of bias graph.
