# Supplementary material for: Effectiveness and Safety of Acupuncture and Moxibustion for Primary Dysmenorrhea: An Overview of Systematic Reviews and Meta-Analyses
Source: Evid Based Complement Alternat Med. 2020 Apr 29;2020:8306165. doi: 10.1155/2020/8306165 (PMC7206866; doi:10.1155/2020/8306165)
Supplement: Supplementary Materials — Additional file 1: exclusion list; additional file 2: AMSTAR 2 evaluation of acupuncture and moxibustion for PD; additional file 3: PRISMA evaluation of acupuncture and moxibustion for PD; additional file 4: risk of bias summary; additional file 5: risk of bias graph. [file 8306165.f1.doc]

**Additional file 1:Exclusion list**

**Here are the reasons for 18 publications’ exclusion during the full-text screening process:13 records were not interested of the research, 3 papers with on access to get the full-text, 2 citations were not interested of the intervention(Acupuncture were not the main intervention in study).**

| **reviews** | **Reason for exclusion** |
| --- | --- |
| Armour M, Smith CA, Steel KA et al., "The effectiveness of self-care and lifestyle interventions in primary dysmenorrhea: a systematic review and meta-analysis," BMC Complement Altern Med, vol. 19, no. 01, pp 22, 2019. | not the intervention of interest |
| Dunselman GAJ, Vermeulen N, Becker C et al., "ESHRE guideline: Management of women with endometriosis," HUM REPROD, vol. 29, no. 03, pp 400-412, 2014. | not the research of interest |
| Gholami Z, "The primary dysmenorrhea and complementary medicine in Iran: A systematic review," International Journal of Fertility and Sterility, vol. 9, pp 109, 2015. | no access to get the full-text |
| Gilbey A, Ernst E, Tani K, "A systematic review of reviews of systematic reviews of acupuncture," Focus on Alternative and Complementary Therapies, vol. 18, no. 01, pp 8-18, 2013. | not the research of interest |
| Jiang HR, Ni S, Li JL et al., "Systematic review of randomized clinical trials of acupressure therapy for primary dysmenorrhea," Evidence-based Complementary and Alternative Medicine, 2013. | not the research of interest |
| Kannan P, Claydon LS, "Some physiotherapy treatments may relieve menstrual pain in women with primary dysmenorrhea: a systematic review," J Physiother, vol. 60, no. 01, pp 13-21, 2014. | not the research of interest |
| Li G, Si J, Zhao C et al., "Network meta-analysis on clinical effects of acupuncture in treatment of primary dysmenorrhea," Chinese Journal of Evidence-Based Medicine, vol. 17, no. 10, pp 1212-1223, 2017. | network meta analysis |
| Proctor ML, Smith CA, Farquhar CM et al., "Transcutaneous electrical nerve stimulation and acupuncture for primary dysmenorrhoea," Cochrane Database Syst Rev, vol. 1, D2123, 2002. | no access to get the full-text |
| Selva OA, Martínez ZMJ, Solà I et al., "Efficacy and safety of needle acupuncture for treating gynecologic and obstetric disorders: An overview," Medical Acupuncture, vol. 25, no. 06, pp 386-397, 2013. | not the research of interest |
| Sharghi M, Mansurkhani SM, Ashtary-Larky D et al., "An update and systematic review on the treatment of primary dysmenorrhea," Jornal Brasileiro de Reproducao Assistida, vol. 23, no. 1, pp 51-57, 2019. | not the intervention of interest |
| Smith CA, Zhu X, He L et al., "Acupuncture for primary dysmenorrhoea," Cochrane Database Syst Rev, vol. 1, D7854, 2011. | no access to get the full-text |
| Song HJ, Seo HJ, Lee H et al., "Effect of self-acupressure for symptom management: A systematic review," Complementary Therapies in Medicine, vol. 23, no. 01, pp 68-78, 2015. | not the research of interest |
| Sun J, Wang Y, Zhang Z et al., "Efficacy of filiform needle manipulation on primary dysmenorrhea:a systematic review," Zhongguo Zhen Jiu, vol. 37, no. 08, pp 887-892, 2017. | one paper has been published in different language |
| Taraqi ASM., "Efficacy of auricular therapy for endometriosis: A review study," Journal of Reproduction and Infertility, vol. 18, no. 02, pp 138-139, 2017. | not the research of interest |
| Wang Y, Sun J, Zhang Z et al., "Impact of deqi on acupoint effects in patients with primary dysmenorrhea:a systematic review of randomized controlled trials," Zhongguo Zhen Jiu, vol. 37, no. 07, pp 791-797, 2017. | not the research of interest |
| Yu S, Yang J, Yang M et al., "Application of acupoints and meridians for the treatment of primary dysmenorrhea: A data mining-based literature study," Evidence-Based Medicine, 2015. | not the research of interest |
| Zhang F, Sun M, Han S et al., "Acupuncture for Primary Dysmenorrhea: An Overview of Systematic Reviews," Evidence-Based Complementary and Alternative Medicine, 2018. | not the research of interest |
| Zhu X, Hamilton KD, McNicol ED., "Acupuncture for pain in endometriosis," Cochrane Database Syst Rev, vol. 9, D7864, 2011. | not the research of interest |
| Gou Chaoqin, Gao Jing, Wu Chenxi et al., "Meta-analysis on the effect of moxibustion quantity and time parameters of moxibustion on primary dysmenorrhea," Clinical Research of Chinese Medicine, vol. 8, no. 34, pp 14-20, 2016. | an article has been published more than once |
| Rao Ying, Li Lei, Liu Xu et al., "Meta-analysis of the clinical effect of acupuncture and moxibustion on primary dysmenorrhea," 11th academic exchange conference of sichuan physiological science society, pp. 31-32. | conference |
| Wang Yafeng, "Meta-analysis on the treatment of primary dysmenorrhea with acupuncture and sanyin crossing," The 4th international acupuncture and massage technique demonstration and 2016 academic annual conference on application of acupoints and acupuncture education, p2, 2016. | conference |
| Xiong Jun, Chen Rixin, Zou Jun et al., “Multi-evaluation of literature quality based on systematic evaluation/meta-analysis of acupuncture and moxibustion for primary dysmenorrhea,”  References | not the research of interest |
| Feng Zhijie, “Meta-analysis of the clinical efficacy of simple acupuncture in treating primary dysmenorrhea,” Inner Mongolia Medical University, 2018. | network meta analysis |
| Li Ge, Si Jinhua, Zhao Chen et al., “Meta-analysis of acupuncture therapy for primary dysmenorrhea,” Chinese Journal of Evidence-Based Medicine, vol. 17, no. 10, pp 1212-1223, 2017. | network meta analysis |
| Abaraogu UO, Tabansi-Ochuogu CS, “As Acupressure Decreases Pain, Acupuncture May Improve Some Aspects of Quality of Life for Women with Primary Dysmenorrhea: A Systematic Review with Meta-Analysis,” Journal of Acupuncture and Meridian Studies, vol. 8, no. 05, 2015. | not the research of interest |

**Additional file 2: AMSTAR 2 evaluation of Acupuncture and Moxibustion for PD.**

| **Review** | 1 | 2 | 3 | 4 | 5 | 6 | 7 | 8 | 9 | 10 | 12 | 13 | 14 | 15 | 16 | score | quality |
| --- | --- | --- | --- | --- | --- | --- | --- | --- | --- | --- | --- | --- | --- | --- | --- | --- | --- |
| Fan2012[24] | Y | N | N | P | N | N | N | P | P | N | N | Y | Y | P | N | 6 | critically low |
| Lan2009[25] | Y | N | N | P | Y | Y | N | P | P | Y | N | Y | N | N | N | 6.5 | critically low |
| Chen2019[26] | Y | N | N | P | Y | Y | N | P | Y | N | N | N | Y | P | N | 7.5 | critically low |
| Xu 2019[27] | Y | N | N | P | Y | N | N | P | P | Y | N | Y | Y | P | N | 8 | critically low |
| Wang2018[28] | Y | N | N | P | N | N | N | P | Y | N | N | Y | Y | P | N | 6.5 | critically low |
| Zhou2018[29] | Y | N | N | P | Y | Y | N | P | Y | Y | N | Y | Y | P | N | 8.5 | critically low |
| Lu 2018[30] | Y | N | N | P | Y | Y | N | P | Y | Y | Y | Y | Y | P | Y | 11.5 | Low |
| Hye LinWoo2018[31] | Y | N | N | P | Y | Y | Y | P | Y | Y | N | Y | Y | P | Y | 11.5 | Low |
| Tong, L 2017[32] | Y | N | N | P | Y | Y | Y | P | Y | Y | N | Y | Y | N | Y | 11 | Low |
| Fan 2017[33] | Y | N | N | P | Y | Y | N | P | Y | N | N | N | Y | P | N | 6.5 | critically low |
| Sun 2017[34] | Y | Y | N | P | Y | Y | N | P | P | Y | N | Y | Y | N | P | 9 | critically low |
| Qin 2017[35] | Y | N | N | P | Y | Y | P | P | P | Y | N | Y | Y | P | P | 9 | critically low |
| Gou 2017[36] | Y | N | N | P | Y | Y | N | P | Y | N | N | Y | Y | Y | N | 8 | Low |
| Liu 2016[37] | Y | N | N | P | Y | Y | N | P | P | N | N | Y | Y | P | N | 7 | critically low |
| Gou 2016[38] | Y | N | N | P | Y | Y | N | P | Y | N | N | Y | Y | Y | N | 8 | Low |
| Wang 2016[14] | Y | N | N | P | Y | Y | Y | Y | Y | N | N | Y | Y | Y | N | 9.5 | moderate |
| Lin 2015[39] | Y | N | N | P | Y | Y | N | P | P | N | Y | Y | N | P | N | 7.5 | critically low |
| Qin 2014[40] | Y | N | N | P | Y | Y | N | P | Y | N | N | Y | Y | P | N | 8.5 | Low |
| Listijo, E 2014[41] | Y | N | N | P | Y | Y | N | P | Y | N | Y | N | N | Y | N | 7 | critically low |
| Chen 2013[42] | Y | N | N | P | Y | Y | N | P | P | Y | N | Y | Y | Y | Y | 9.5 | Low |
| Yang 2008[43] | Y | N | N | P | Y | Y | N | P | Y | N | N | Y | Y | N | Y | 8.5 | critically low |
| Chen 2013[44] | Y | N | N | P | Y | Y | N | P | Y | N | N | Y | Y | N | Y | 9 | critically low |
| Yu, S. Y 2017[45] | Y | N | N | P | Y | Y | N | Y | Y | Y | N | Y | N | Y | Y | 9.5 | moderate |
| Xu, Y 2017[13] | Y | N | N | P | Y | Y | N | P | Y | Y | N | Y | Y | P | Y | 9.5 | Low |
| CarolineASmith2016[15] | Y | Y | N | Y | Y | Y | Y | Y | Y | Y | N | Y | Y | Y | Y | 14 | high |
| TianXu,BSc2014[46] | Y | Y | N | P | Y | Y | N | P | Y | Y | N | Y | Y | Y | Y | 12 | moderate |
| Chung, Y.C 2012[47] | Y | N | N | P | Y | Y | N | P | P | N | N | Y | Y | Y | Y | 9.5 | Low |
| Cho, S.H 2010[48] | Y | N | N | P | Y | Y | N | P | Y | N | Y | Y | N | N | Y | 8 | critically low |
| Total score:246.5; Mean score:8.8 | | | | | | | | | | | | | | | | | |

Y: Yes (1point); P: Partial Yes(0.5 point); N: No(0point).

**Additional file 3: PRISMA evaluation of Acupuncture and Moxibustion for PD.**

| **Review** | 1 | 2a | 2b | 2c | 2d | 2e | 2f | 2g | 2h | 2i | 2j | 2k | 3 | 4 | 5 | 6 | 7 | 8 | 9 | 10 |
| --- | --- | --- | --- | --- | --- | --- | --- | --- | --- | --- | --- | --- | --- | --- | --- | --- | --- | --- | --- | --- |
| Fan2012[24] | Y | Y | Y | Y | N | Y | P | Y | N | Y | N | N | Y | Y | N | Y | Y | P | N | N |
| Lan2009[25] | Y | Y | Y | Y | N | N | N | N | N | Y | N | N | Y | Y | N | Y | Y | P | P | N |
| Chen2019[26] | Y | Y | Y | Y | N | Y | P | Y | N | Y | N | N | Y | Y | N | Y | Y | P | P | Y |
| Xu 2019[27] | Y | Y | Y | Y | Y | Y | P | Y | Y | Y | N | N | N | Y | N | Y | Y | P | N | Y |
| Wang2018[28] | Y | Y | Y | Y | N | Y | P | Y | Y | Y | N | N | Y | Y | N | Y | Y | P | Y | Y |
| Zhou2018[29] | Y | Y | Y | Y | Y | Y | P | Y | N | Y | N | N | Y | Y | N | Y | Y | P | Y | Y |
| Lu 2018[30] | Y | Y | Y | Y | N | Y | P | Y | Y | Y | N | N | Y | Y | N | Y | Y | P | Y | Y |
| Hye LinWoo2018[31] | Y | Y | Y | Y | N | Y | P | Y | Y | Y | N | N | Y | Y | Y | Y | Y | P | Y | Y |
| Tong, L 2017[32] | Y | Y | Y | Y | N | Y | P | Y | N | Y | N | N | Y | Y | N | Y | Y | P | Y | P |
| Fan 2017[33] | Y | Y | Y | Y | Y | Y | P | Y | Y | Y | N | N | Y | Y | N | Y | Y | Y | Y | Y |
| Sun 2017[34] | Y | Y | Y | Y | N | Y | N | N | Y | Y | N | N | Y | Y | Y | Y | Y | P | Y | Y |
| Qin 2017[35] | Y | Y | Y | Y | N | N | N | N | Y | Y | N | N | Y | Y | N | Y | Y | P | Y | Y |
| Gou 2017[36] | Y | Y | Y | Y | Y | Y | P | Y | Y | Y | N | N | Y | Y | N | Y | Y | Y | Y | Y |
| Liu 2016[37] | Y | Y | Y | Y | N | Y | P | N | Y | Y | N | N | Y | Y | N | Y | Y | P | Y | Y |
| Gou 2016[38] | Y | Y | Y | Y | Y | Y | P | Y | Y | Y | N | N | Y | Y | N | Y | Y | Y | Y | Y |
| Wang 2016[14] | Y | Y | Y | Y | N | Y | P | Y | Y | Y | N | N | Y | Y | N | Y | Y | Y | Y | Y |
| Lin 2015[39] | Y | Y | Y | Y | N | N | N | N | Y | Y | N | N | Y | Y | N | Y | Y | P | Y | Y |
| Qin 2014[40] | Y | Y | Y | Y | N | Y | P | Y | Y | Y | N | N | Y | Y | N | Y | Y | P | Y | Y |
| Listijo, E 2014[41] | Y | Y | Y | Y | Y | Y | N | N | Y | Y | N | N | Y | Y | N | Y | Y | P | Y | Y |
| Chen 2013[42] | Y | Y | Y | N | N | Y | P | Y | Y | Y | N | N | Y | Y | N | Y | Y | P | Y | Y |
| Yang 2008[43] | Y | Y | Y | Y | Y | Y | Y | Y | Y | Y | N | N | Y | Y | N | Y | Y | Y | Y | Y |
| Chen 2013[44] | Y | Y | Y | Y | N | N | P | Y | Y | Y | N | N | Y | Y | N | Y | Y | Y | Y | Y |
| Yu, S. Y 2017[45] | Y | Y | Y | Y | N | N | N | N | Y | Y | N | N | Y | Y | N | Y | Y | P | P | Y |
| Xu, Y 2017[13] | Y | Y | Y | Y | N | Y | P | Y | N | Y | N | N | Y | Y | N | Y | Y | P | P | Y |
| CarolineASmith2016[15] | Y | Y | Y | Y | N | Y | Y | Y | Y | Y | N | N | Y | Y | Y | Y | Y | Y | Y | Y |
| TianXu,BSc2014[46] | N | Y | Y | Y | N | Y | P | Y | Y | Y | N | N | Y | Y | Y | Y | Y | P | Y | Y |
| Chung, Y.C 2012[47] | Y | Y | Y | Y | N | Y | N | N | Y | Y | N | N | Y | Y | N | Y | Y | P | Y | Y |
| Cho, S.H 2010[48] | Y | Y | Y | Y | N | Y | N | N | Y | Y | N | N | Y | Y | N | P | Y | P | Y | Y |

| **Review** | 11 | 12 | 13 | 14 | 15 | 16 | 17 | 18 | 19 | 20 | 21 | 22 | 23 | 24 | 25 | 26 | 27 | score |
| --- | --- | --- | --- | --- | --- | --- | --- | --- | --- | --- | --- | --- | --- | --- | --- | --- | --- | --- |
| Fan2012[24] | Y | Y | Y | Y | Y | Y | Y | Y | Y | Y | Y | Y | Y | P | Y | Y | N | 27.5 |
| Lan2009[25] | N | P | Y | N | N | Y | N | Y | Y | Y | Y | Y | Y | P | Y | P | P | 21.5 |
| Chen2019[26] | Y | Y | Y | Y | Y | N | Y | Y | Y | Y | Y | Y | N | P | N | N | N | 25.5 |
| Xu 2019[27] | N | Y | Y | Y | Y | Y | Y | Y | Y | Y | Y | Y | N | P | Y | Y | P | 28 |
| Wang2018[28] | Y | P | Y | Y | Y | Y | Y | Y | Y | Y | Y | Y | Y | P | Y | Y | N | 30 |
| Zhou2018[29] | N | Y | Y | Y | Y | N | Y | Y | N | Y | Y | Y | N | P | Y | Y | P | 27 |
| Lu 2018[30] | N | P | Y | Y | Y | N | Y | Y | Y | Y | Y | Y | Y | P | Y | Y | P | 28.5 |
| Hye LinWoo2018[31] | Y | Y | Y | Y | Y | Y | Y | Y | Y | Y | Y | Y | Y | P | Y | Y | Y | 32.5 |
| Tong, L 2017[32] | Y | P | Y | Y | P | Y | Y | Y | Y | Y | Y | Y | Y | Y | Y | Y | Y | 29 |
| Fan 2017[33] | Y | P | Y | Y | Y | N | Y | Y | Y | Y | Y | Y | Y | P | Y | Y | N | 30.5 |
| Sun 2017[34] | Y | Y | Y | Y | N | N | Y | Y | Y | Y | P | Y | N | P | Y | Y | P | 27.5 |
| Qin 2017[35] | Y | Y | Y | Y | Y | N | Y | Y | Y | Y | Y | Y | N | P | Y | Y | P | 26.5 |
| Gou 2017[36] | Y | P | Y | Y | Y | Y | Y | Y | Y | Y | Y | Y | N | P | Y | Y | N | 30.5 |
| Liu 2016[37] | Y | P | Y | Y | Y | N | Y | Y | Y | Y | Y | Y | N | P | N | Y | N | 26 |
| Gou 2016[38] | Y | P | Y | Y | Y | Y | Y | Y | Y | Y | Y | Y | N | P | Y | Y | N | 30.5 |
| Wang 2016[14] | Y | Y | Y | Y | Y | Y | Y | Y | Y | Y | Y | Y | Y | P | Y | Y | N | 31 |
| Lin 2015[39] | N | Y | Y | Y | P | N | Y | Y | Y | Y | Y | Y | N | P | Y | Y | N | 24.5 |
| Qin 2014[40] | N | P | Y | Y | Y | N | Y | Y | Y | Y | Y | Y | Y | P | Y | Y | N | 28 |
| Listijo, E 2014[41] | Y | P | Y | Y | Y | Y | Y | Y | Y | Y | Y | Y | Y | P | Y | Y | N | 29.5 |
| Chen 2013[42] | Y | P | Y | Y | Y | Y | Y | Y | Y | Y | Y | Y | N | P | Y | Y | Y | 29 |
| Yang 2008[43] | Y | Y | Y | N | N | N | Y | Y | Y | Y | N | Y | N | P | Y | Y | Y | 28.5 |
| Chen 2013[44] | P | P | Y | Y | P | Y | Y | Y | Y | Y | Y | Y | Y | Y | Y | Y | Y | 29.5 |
| Yu, S. Y 2017[45] | Y | P | Y | Y | Y | Y | Y | Y | Y | Y | Y | Y | Y | Y | Y | Y | Y | 28.5 |
| Xu, Y 2017[13] | Y | P | Y | Y | Y | Y | Y | Y | Y | Y | Y | Y | N | Y | Y | Y | Y | 29 |
| CarolineASmith2016[15] | Y | Y | Y | Y | Y | Y | Y | Y | Y | Y | Y | Y | Y | Y | Y | Y | Y | 33 |
| TianXu,BSc2014[46] | N | Y | Y | Y | Y | Y | Y | Y | Y | Y | Y | Y | Y | Y | Y | Y | Y | 32 |
| Chung, Y.C 2012[47] | Y | P | Y | Y | Y | Y | Y | Y | Y | Y | Y | Y | Y | Y | Y | Y | N | 29 |
| Cho, S.H 2010[48] | P | Y | Y | N | P | N | Y | P | Y | Y | Y | P | N | Y | Y | Y | Y | 24 |
| Total score:796.5; Mean score:28.45 | | | | | | | | | | | | | | | | | | |

Y: Yes (1point); P: Partial Yes(0.5 point); N: No(0point).

**Additional file 4: Risk of bias summary**


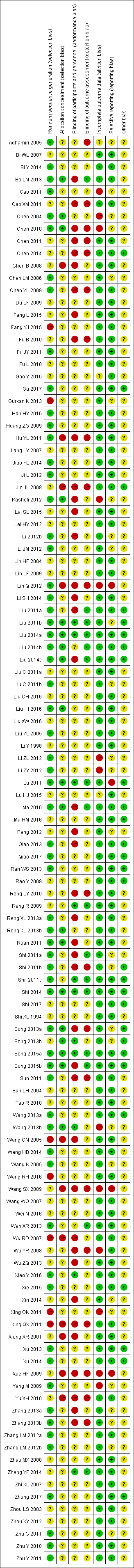

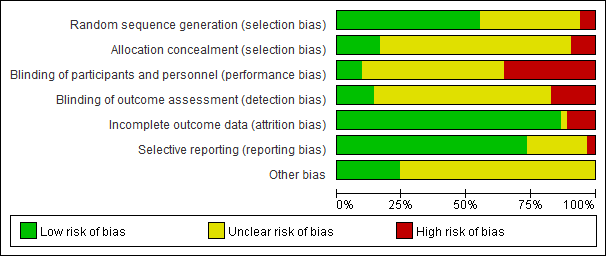


**Additional file 5:Risk of bias graph**
